# Supplementary figures and images for: A Heterochromatic Knob Reducing the Flowering Time in Maize
Source: Front Genet. 2022 Feb 24;12:799681. doi: 10.3389/fgene.2021.799681 (PMC8908004; doi:10.3389/fgene.2021.799681)

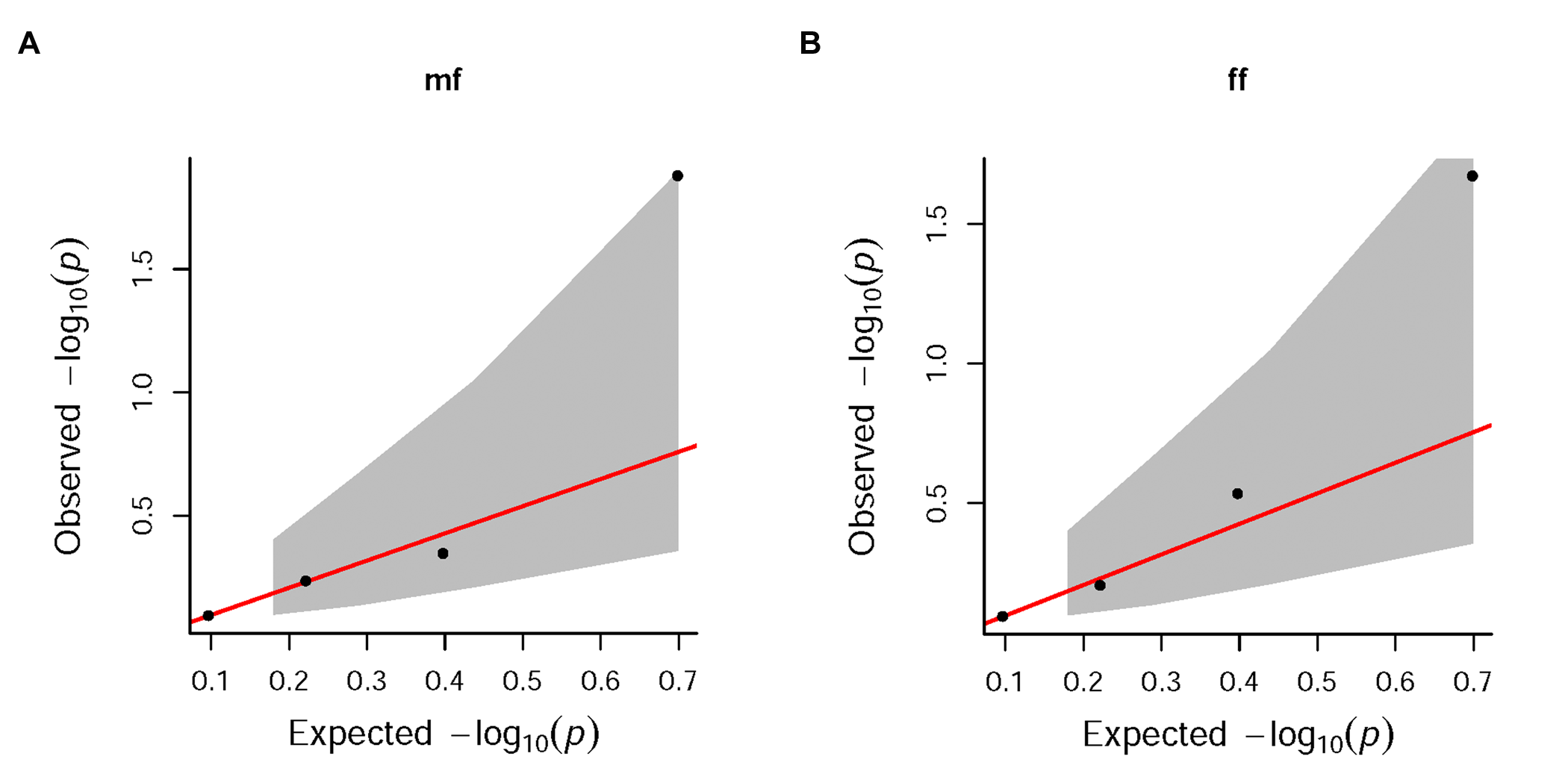

Supplement: Supplementary file 2 [file Image3.TIF]

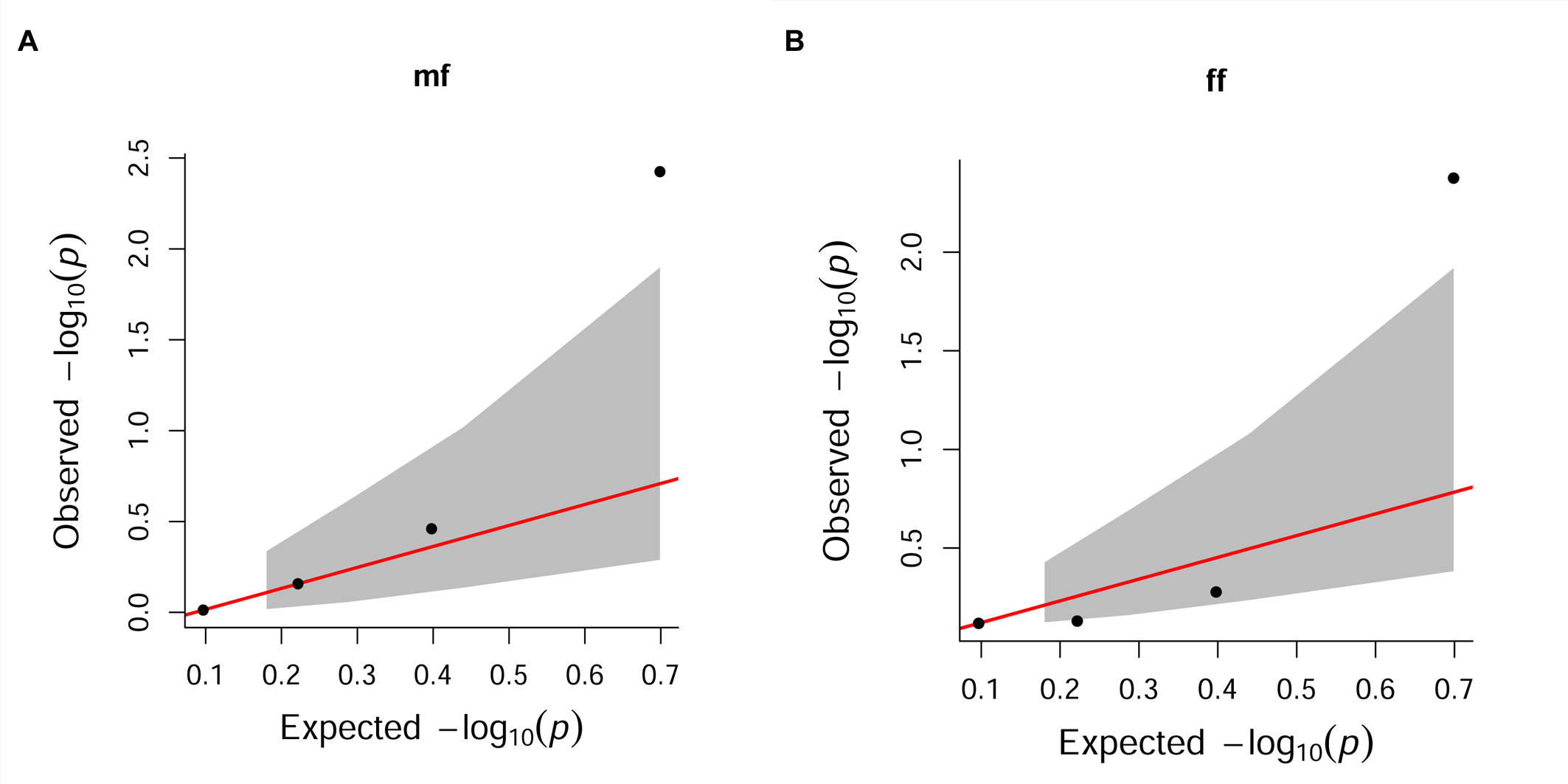

Supplement: Supplementary file 3 [file Image4.TIF]

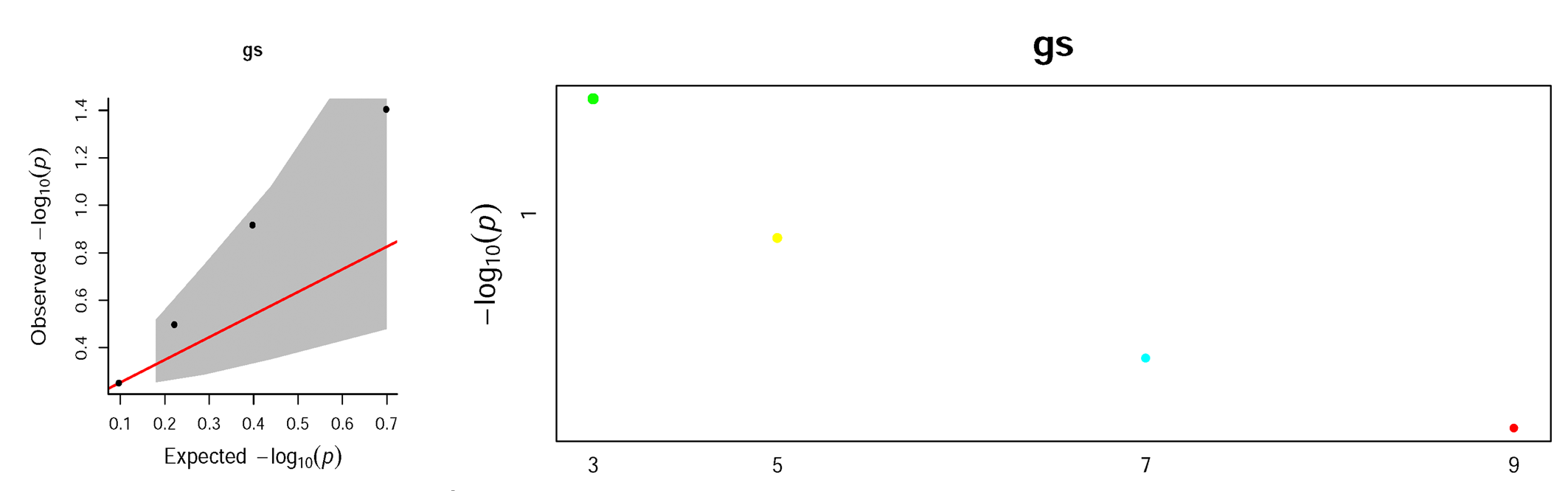

Supplement: Supplementary file 4 [file Image2.TIF]

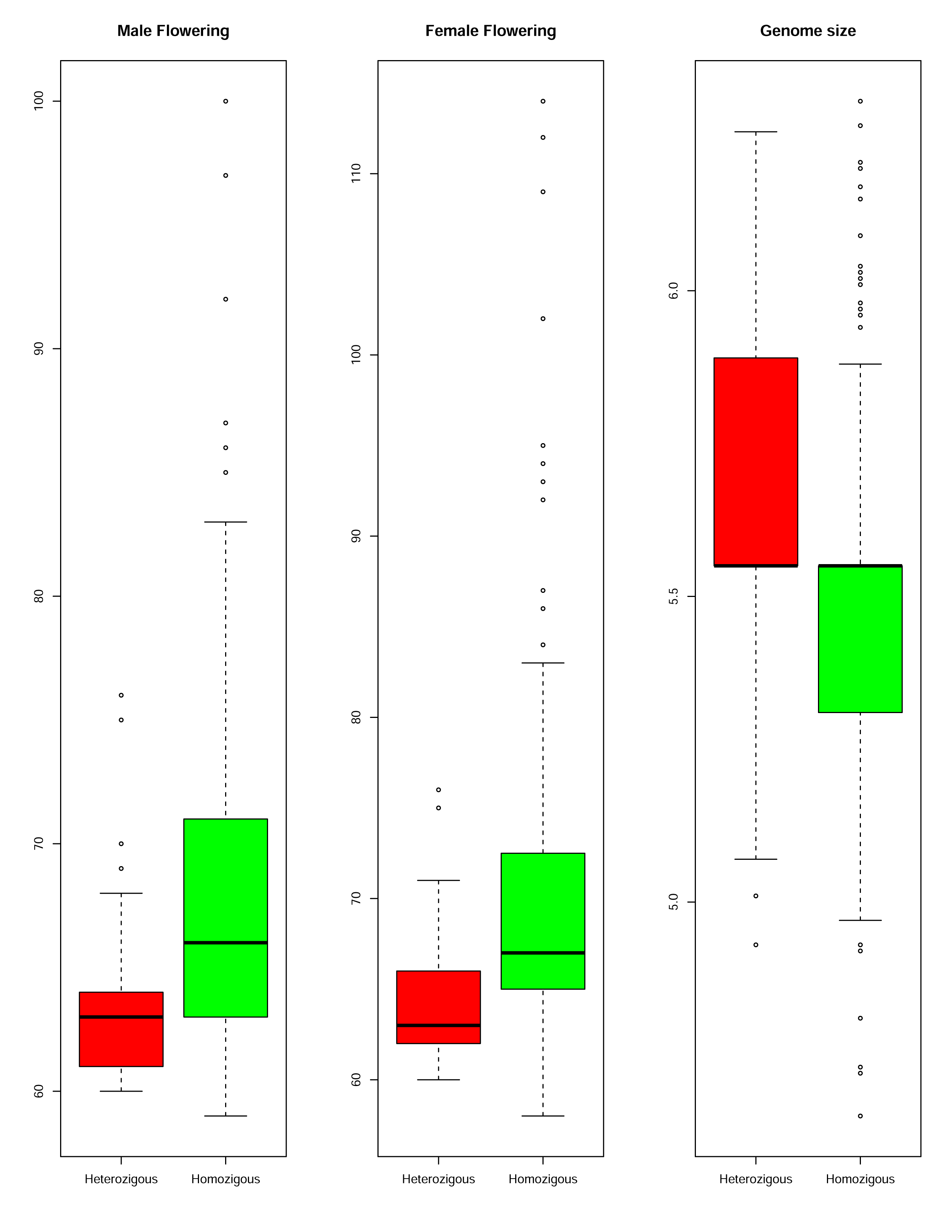

Supplement: Supplementary file 5 [file Image1.TIF]
